# A novel Bcl-2 inhibitor, BM-1197, induces apoptosis in malignant lymphoma cells through the endogenous apoptotic pathway

**DOI:** 10.1186/s12885-019-6169-0

**Published:** 2019-12-31

**Authors:** Yue-Li Sun, Wen-Qi Jiang, Qiu-Yun Luo, Da-Jun Yang, Yu-Chen Cai, Hui-Qiang Huang, Jian Sun

**Affiliations:** 1 Guangdong Lung Cancer Institute, Guangdong Provincial People’s Hospital and Guangdong Academy of Medical Sciences, Guangzhou, 510080 China; 2 State Key Laboratory of Oncology in South China, Collaborative Innovation Center for Cancer Medicine, Sun Yat-sen University Cancer Center, Guangzhou, 510060 China; 3 Department of Medical Oncology, Sun Yat-sen University Cancer Center, Guangzhou, 510060 Guangdong China; 4 Department of Experimental Research, Sun Yat-sen University Cancer Center, Guangzhou, 510060 China; 5 Department of Clinical Research, Sun Yat-sen University Cancer Center, 651 Dongfeng East Road, Guangzhou, 510060 China

**Keywords:** Bcl-2 inhibitor, Malignant lymphoma, Apoptosis, Targeted therapy, Chemotherapy

## Abstract

**Background:**

Bcl-2 family members play an important role in the development of malignant lymphoma and can induce drug resistance in anticancer treatment. The development of small molecules targeting Bcl-2 family proteins may be a new strategy for the treatment of malignant lymphoma. In this study, we investigate the antitumor effect and cellular mechanism of a novel Bcl-2/Bcl-xL dual inhibitor, BM-1197, in DCBCL and Burkitt lymphoma cells.

**Methods:**

The CCK-8 assay was used to detect cell viability. Apoptosis was determined by Hoechst 33258 staining and flow cytometry. The activity of caspase-3/caspase-9 was determined using a caspase-3/caspase-9 activity kit. Western blotting analysis was performed to evaluate the changes in protein expression. Functional analysis was performed via immunoprecipitation and siRNA interference. Human malignant lymphoma xenograft models in nude mice were established for in vivo efficacy detection.

**Results:**

We find that BM-1197 exerts potent growth-inhibitory activity against lymphoma cells that harbor high expression of Bcl-2 and Bcl-xL in vitro and has a synergistic effect with chemotherapeutic drugs. Mechanistically, we see that the intrinsic apoptosis pathway is activated upon BM-1197 treatment. BM-1197 affects the protein interactions of Bak/Bcl-xl, Bim/Bcl-2, Bim/Bcl-xl, and PUMA/Bcl-2 and induces conformational changes in the Bax protein, which result in the activation of Bax and release of cytochrome c, activate caspase − 9, − 3, and − 7 and finally induce cell apoptosis. Furthermore, our data demonstrate that BM-1197 exhibits strong anti-tumor effects against established human malignant lymphoma xenograft models.

**Conclusions:**

Our study demonstrated BM-1197 exerts potent antitumor effects both in vitro and in vivo and provides promising preclinical data for the further development of BM-1197 in malignant lymphoma.

## Background

Diffuse large B-cell lymphoma (DLBCL) is the most common lymphoid malignancy in adults, accounting for 30–40% of all non-Hodgkin’s lymphoma (NHL) [1]. Although approximately 60% of patients with DLBCL can be cured by using the standard immunochemotherapy regimen, rituximab combined with cyclophosphamide, doxorubicin, vincristine, and prednisone (R-CHOP), the remaining patients are either refractory or relapse after achieving complete remission [2, 3]. About one third of patients relapse after complete response to the first-line therapy [2, 4]. Only ~ 10% of refractory or relapsed patients can be cured with conventional salvage immunochemotherapy with autologous transplant [5, 6]. The majority have very dismal outcomes, warranting the development of novel therapeutic approaches.

Bcl-2 is upregulated by translocation or other mechanisms, including Bcl-2 gain/amplification, in approximately 50% of DLBCL [7]. The Bcl-2 protein promotes the survival of cancer cells by inhibiting apoptosis, and it also induces resistance to chemotherapy. High Bcl-2 expression was an adverse prognostic factor independent of IPI in the prerituximab era [8]. Although the prognostic role of isolated Bcl-2 overexpression has been diminished by the addition of rituximab [9], it remains significant in activated B-cell (ABC) subtype disease. ABC DLBCL is more commonly mediated by Bcl-2 gain amplification and is associated with inferior PFS [10]. Bcl-2 inhibition is therefore an attractive therapeutic target for B cell lymphoma.

Bcl-2 family proteins play their biological role through Bcl-2 homology domains (BH domains), which are the core and structural basis of protein interactions and are essential for pro-apoptotic activity [11]. BM-1197, a small molecular compound structurally similar to BH3, has a high binding capacity with Bcl-2 family proteins. The antitumor activity of BM-1197 was induced by apoptosis in lung cancer cells. It also shows good antitumor activity in a mouse lung cancer xenograft model [12]. BM-1197 is therefore a promising drug candidate for cancer therapy. The aim of this study is to explore the anti-tumor effect of BM-1197 in non-Hodgkin’s lymphoma and its combined application with common chemotherapeutic drugs.

## Methods

### Chemicals

BM-1197 was kindly provided by Professor Yang Dajun.BEZ235 and ABT-263 were purchased from Selleckchem (Houston, TX). Gemcitabine hydrochloride was purchased from Jiangsu Haosen Company and Doxorubicin hydrochloride was purchased from Pharmacia Co., Ltd. Vinblastine sulfate was purchased from Shenzhen Wanle Pharmaceutical Co., Ltd. For in vitro assays, all compounds were dissolved in dimethylsulfoxide (DMSO; Sigma Aldrich, MO, USA) at a stock concentration of 40 mM and stored at − 20 °C. The final concentration of DMSO to dilute compound in culture media did not exceed 0.1%.

### Cell lines and cell culture

The cell lines used in this study include OCI-ly1, OCI-ly8, OCI-ly19, Su-DHL-4 and Nu-DHL-1 cells, which are from diffuse large B cell lymphoma. The first three cell lines were kindly provided by Ru feng in Nanfang hospital, Guangzhou, Guangdong in 2006. The last two cell lines were kindly supported by Yanhui Liu in Guangdong Provincial People’s Hospital in 2007. Raji, Ramos and Namalwa cells are from Burkitt lymphoma and these cell lines were frozen in liquid nitrogen in our lab. Jurkat cells are from human peripheral blood leukemia T cells. The cell lines were identified by genomic short tandem repeat (STR) profile detection and have been tested for no mycoplasma contamination. All cells were cultured at 37 °C with 5% CO_2_. OCI-ly1, OCI-ly8, OCI-ly19, Raji, Ramos, and Namalwa cells were cultured in RPMI-1640 medium containing 10% fetal bovine serum, while Su-DHL-4 and Nu-DHL-1 cells were cultured in RPMI-1640 medium containing 20% ​​fetal bovine serum. All culture media contained 100 IU/ml penicillin and 100 mg/ml streptomycin. All experiments were performed in the logarithmic growth phase of the cells.

### Cell viability assay

Cell viability was determined by a CCK-8 kit. Lymphoma cells at the logarithmic growth phase were inoculated into 96-well culture plates with 180 μl of medium (cell concentration: 5000/well). After incubation for 24 h, 20 μl of BM-1197 at different concentration was added. After incubation for 72 h, 10 μl of CCK-8 was added to each well and incubated for 3–4 h. OD values were measured at 450 nm using a microplate reader. All experiments were performed in triplicate.

The combination effect of BM-1197 and other chemotherapy drugs were determined by combined index value (CI value). After OCI-ly8 cells incubation for 24 h, BM-1197 was combined with Adriamycin, Gemcitabine and Vincristine at indicated ratio and concentration. After incubation for 68 h, 10 μl of CCK-8 was added to each well and incubated for another 3–4 h. The OD value was read at 450 nm using a microplate reader. The inhibition rate was calculated according to the above formula. CompuSyn software (Chou-Talalay formula) was used to calculate the combined index value (CI value). A CI value less than 0.1 indicated a strong synergistic effect of two drugs; a CI value less than 0.9 indicated a synergistic effect of two drugs; a CI value greater than 0.9 and less than 1.1 indicated a weak synergistic effect; and a CI values greater than 1.1 indicated an antagonistic effect of two drugs.

### Hoechst 33258 staining for the detection of apoptotic morphological changes

OCI-ly8 cells were treated with BM-1197 at different concentrations (0, 0.5, 1.0, 2.0 μM) for 24 h. The cells were collected and fixed with 0.5 ml of fixative at 4 °C overnight. After fixation, cells were washed twice with PBS, each for 3 min. Cells were fixed on a glass slide, and 0.5 ml of Hoechst 33258 staining solution was added for 5 min. After washing in PBS, anti- quenching solution was added, followed by cover slipping. Blue nuclear staining was observed under a fluorescence microscope with an excitation wavelength of approximately 350 nm and an, emission wavelength of approximately 460 nm.

### Annexin V / PI double staining for the detection of early apoptosis

OCI-ly8 cells in the logarithmic growth phase were inoculated in 6-well plates at 3 × 105 per well and incubated for 2–4 h. After treatment with BM-1197 at different concentrations (0, 0.5, 1.0, 2.0 μM) for 24 h, the cells were collected. Cells were washed with PBS twice, and 20 μl of Annexin-V and PI staining solution was added. Cells were incubated for 15 min at room temperature in the dark. Stained cells were analyzed within 1 h with a flow cytometer. The negative control group was divided into three groups with binding buffer: one was treated with Annexin V, another was single-stained for PI, and the third was used as a standard sample for flow cytometry.

### Detection of Caspase-3/ caspase-9activity

The activity of caspase-3/caspase-9 was determined using a Caspase-3/caspase-9 activity kit (Beyotime Institute of Biotechnology, Haimen, China). After treatment with BM-1197, cell lysates were prepared by incubating 2 × 106 cells/ml in extraction buffer (25 mM Tris–HCl, pH 7.5, 20 mM MgCl2, 150 mM NaCl, 1% Triton X-100, 25 μg/ml leupeptin, and 25 μg/ml aprotinin) for 30 min on ice. Lysates were centrifuged at 12,000×g for 15 min, the supernatants were collected and protein concentrations were determined by the Bradford Protein Assay Kit (Beyotime Institute of Biotechnology, Haimen, China). Cellular extracts (30 μg) were then incubated in a 96-well plate with 20 ng of Ac-DEVD-pNA/Ac-LEHD-pNA for 2 h at 37 °C. Caspase-3 and caspase-9 activities were measured by cleavage of the Ac-DEVD-pNA and Ac-LEHD-pNA substrates, respectively, to pNA, the absorbance of which was measured at 405 nm. Relative caspase activity was calculated as a ratio of the emission of treated cells to untreated cells.

### Western blot analysis

OCI-ly8 cells were treated with different concentrations of BM-1197 (0, 0.25, 0.5, 1, 2 μM) for 24 h or with 2 μM BM-1197 for 0, 1, 3, 6, 12 and 24 h. After that, cells were collected and washed twice with cold PBS and lysed in 1 × cell lysis buffer, which was diluted from 10 × cell lysis buffer (Cell Signaling Technology). Then, 1x proteinase inhibitor cocktail was added in the lysis buffer. Lysates were centrifuged at 12000 g at 4 °C for 20 min. Supernatants were collected and stored at − 80 °C until used. The protein concentration of the supernatants was determined using BCA protein assay reagents. The relevant primary antibodies used included: Bcl-2, Bax (6A7) and PARP-1 polyclonal antibodies purchased from Santa Cruz Biotechnology, Inc. (Santa Cruz, CA), and anti-Mcl-1, PUMA, Bcl-xl, caspase 3, Caspase 9, cytochrome c (cyt c), and GAPDH monoclonal antibody purchased from Cell Signaling Technology (Danvers, MA). The COXIV polyclonal antibody was purchased from Biyun Tian Biotechnology Research Institute. Mouse and rabbit secondary antibodies were from Cell Signaling Technology (Danvers, MA). Antigen-antibody complexes were detected using the PhototopeTM-HRP Chemiluminescent Substrate System (Cell Signaling Technology) per the manufacturer’s instructions.

### Mitochondrial cytochrome c release assay

OCI-ly8 cells were treated with different concentrations of BM-1197 (0, 0.25, 0.5, 1, 2 μM) for 24 h or with 2 μM BM-1197 for 0, 1, 3, 6, 12 and 24 h. After that, cells were collected and washed twice with cold PBS and lysed in 1 × cell lysis buffer, which was diluted from 10 × cell lysis buffer (Cell Signaling Technology). Then, 1x proteinase inhibitor cocktail was added in the lysis buffer. Lysates were centrifuged at 12000 g at 4 °C for 20 min. Supernatants were collected and stored at − 80 °C until used. The protein concentration of the supernatants was determined using BCA protein assay reagents. The relevant primary antibodies used included: Bcl-2, Bax (6A7) and PARP-1 polyclonal antibodies purchased from Santa Cruz Biotechnology, Inc. (Santa Cruz, CA), and anti-Mcl-1, PUMA, Bcl-xl, caspase 3, Caspase 9, cytochrome c (cyt c), and GAPDH monoclonal antibody purchased from Cell Signaling Technology (Danvers, MA). The COXIV polyclonal antibody was purchased from Biyun Tian Biotechnology Research Institute. Mouse and rabbit secondary antibodies were from Cell Signaling Technology (Danvers, MA). Antigen-antibody complexes were detected using the PhototopeTM-HRP Chemiluminescent Substrate System (Cell Signaling Technology) per the manufacturer’s instructions.

### Immunoprecipitation

OCI-ly8 cells were incubated with 2 μM BM-1197 or 0.1% DMSO for 6 h. The cells (1 × 107) were then harvested and lysed in 500 μl of CHAPS lysis buffer (50 mM Tris-Cl [pH 7.4], 150 mM NaCl, 1% CHAPS, 1 mM EDTA, 1 mM EGTA, protease inhibitors, PhosSTOP [Roche], and 20 mM MG132) on ice for 30 min. Whole cell lysates were obtained, precleared with protein A/G-Sepharose, and incubated overnight with 1 μg of a specific antibody (Bak, Bax, Bcl-2, Bcl-xl, PUMA, Bim) at 4 °C. Immunocomplexes were captured with either protein A-Sepharose or protein G-Agarose. The beads were pelleted, washed 3 times, and boiled in SDS sample buffer. The presence of immunocomplexes was determined by western blot analysis.

### Gene knockdown using siRNA

The siRNAs to Mcl-1 or control siRNA were all purchased from GenePharma (Shanghai). OCI-Ly8 cells were transfected with siRNA using Lipofectamine 2000 (Invitrogen) according to the manufacturer’s instructions. Cells were incubated for 8 h, and then the culture medium was refreshed for further treatment.

### In vivo xenograft studies

In the xenograft cancer model, male nonobese diabetic severe combined immunodeficiency (NOD/SCID) mice were purchased from Charles River (Beijing, China), housed in groups of five and given three days to acclimate to the housing facility. Each of the mice was approximately 4 weeks with a body weight of approximately 18 g before the cancer cells were implanted. The mice were fed under constant temperature (21 °C ± 2 °C) and humidity (55 ± 10%) in specific pathogen free (SPF) rooms in Sun Yat-sen University laboratory animal center with a 12:12 light:dark cycle with lights on at 07:00 and off at 19:00. During housing, animals were monitored twice daily for health status. No adverse events were observed. All the materials were autoclave-sterilized before contacting the mice.

OCI-Ly8 cells were implanted subcutaneously in the right side of axillary with 1 × 10^7^ tumor cells suspended in a 200-μl volume of PBS. For drug efficacy studies, when the mean tumor volume reached approximately 100~200 mm^3^, mice were randomized into the vehicle control group (1% Klucel LF/0.1% Tween 80) or BM-1197 treatment group (10 mg/kg; QOD), with five mice per group. The randomization was performed with a randomized table. Tail intravenous injection was performed in both groups. Tumor sizes were measured by caliper equipment, and animal body weights were recorded 2~3 times per week. Tumor volume (mm^3^) =1/2 × (length×width^2^). At the end of animal experiments, the mice were killed by the cervical dislocation method. All animal experiments were carried out under the guide of the Sun Yat-sen University Committee for the Use and Care of Laboratory Animals and approved by the animal experimentation ethics committee.

### Statistical analysis

All experiments were performed at least three times. Statistical analysis was carried out using Microsoft Excel 2001, and data are presented as the mean ± standard error of the mean (SEM) combining three experimental repeats. *P* < 0.05 was considered a significant difference.

## Results

### High expression level of Bcl-2 predicts sensitivity to BM-1197

**Fig. 1:**
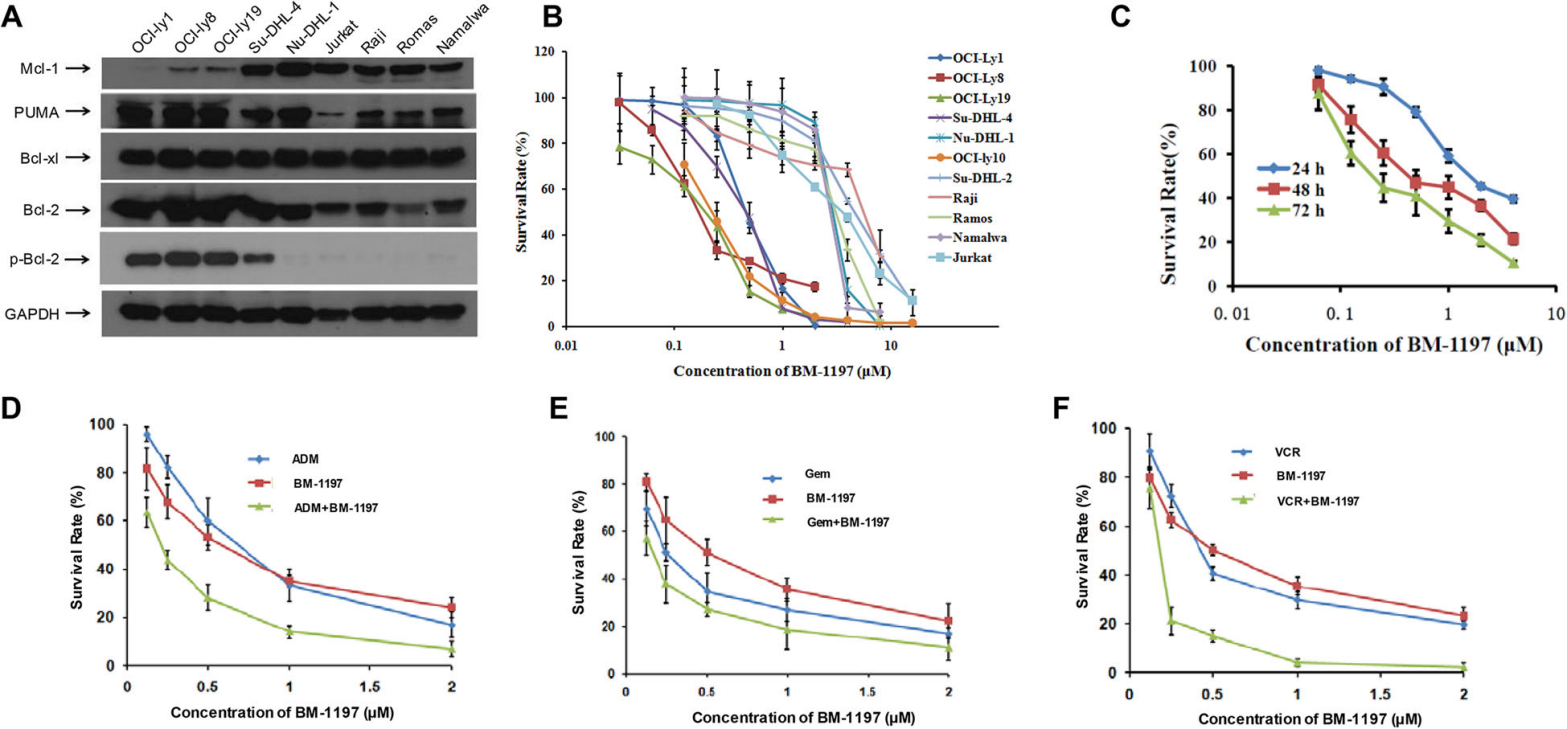
High expression level of BCL-2 predicts sensitivity to BM1197. **a**. Basal protein expression of Bcl-2 family protein in lymphoma cell lines. **b**. BM-1197 inhibits the proliferation of nine lymphoma cell lines in a dose-dependent manner. **c**. The inhibition of BM-1197 on OCI-ly8 cells is in a time-dependent way. **d**. The combination curve of BM-1197 with ADM inOCI-ly8 cells. **e**. The combination curve of BM-1197 with GEM inOCI-ly8 cells. **f**. The combination curve of BM-1197 with VCR inOCI-ly8 cells

The expression of Bcl-2 family proteins was examined in eight different B cell-derived lymphoma cell lines and one T-cell-derived acute T-cell leukemia cell line. As shown in Fig. 1a, Bcl-2 was highly expressed in diffuse large B-cell lymphoma cell lines and expressed at low levels in Burkitt and Jurkat cells. Then, we detected the inhibitory effect of BM-1197 on these lymphoma cells, and we examined cell growth after treatment with BM-1197 using the CCK-8 essay. The IC50 of BM-1197 in each cell line is shown in Table 1. The results showed that BM-1197 was sensitive to OCI-ly1, OCI-ly8 and OCI-ly19 cells, with IC50 values ranging from 0.2 to 0.5 μM (Fig. 1b). After treatment with BM-1197 for 24 h, 48 h, and 72 h, OCI-ly8 cell viability was determined. The results indicated that the cell inhibitory effect of BM-1197 was time-dependent (Fig. 1c). To evaluate whether there is a synergistic effect of BM-1197 with other chemotherapeutic drugs, we determined the growth inhibition of BM-1197 combined with common chemotherapeutic agents such as doxorubicin, vincristine and gemcitabine using the CCK-8 essay in OCI-ly8 cells. The synergistic effects of BM-1197 combined with three chemotherapeutic drugs were demonstrated in Fig. 1d, e, f.

**Table 1:** The IC50 values (μM) of BM-1197 in NHL cell lines

Cell line	IC_50_ (μmol/L)
OCI-ly1	0.47 ± 0.06
OCI-Ly8	0.19 ± 0.04
OCI-ly19	0.22 ± 0.03
Su-DHL-4	0.72 ± 0.16
Nu-DHL-1	2.99 ± 0.36
Raji	5.94 ± 0.04
Romas	3.86 ± 0.99
Namalwa	3.26 ± 0.5
Jurkat	2.16 ± 0.4

Data are the mean ± SEM of three independent experiments

### BM-1197 induced dose- and time-dependent apoptosis

**Fig. 2:**
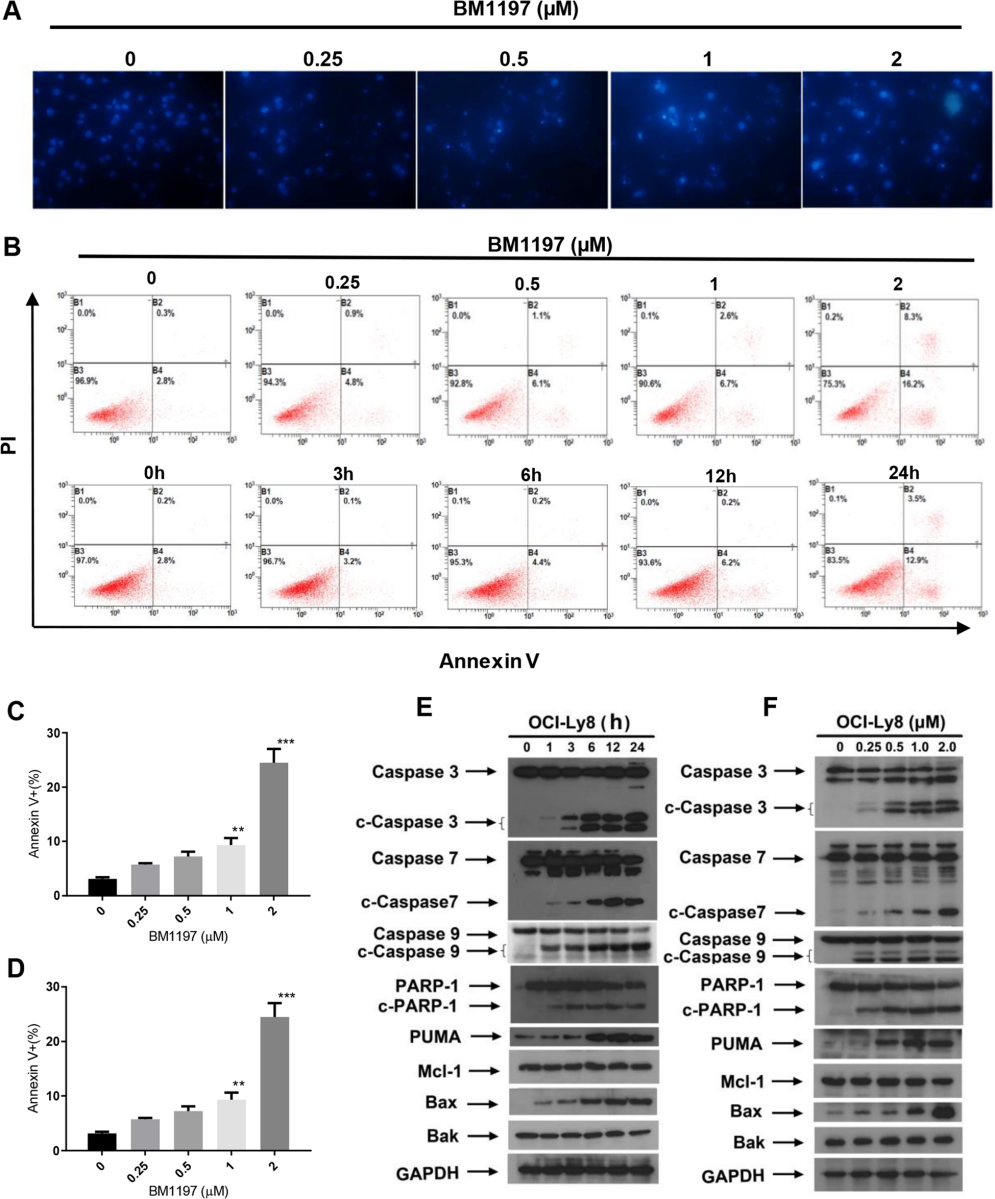
BM-1197 induced dose- and time-dependent apoptosis. **a**. After treatment with different concentrations of BM-1197, apoptotic bodies with intense blue fluorescence (mainly apoptotic cells due to chromatin condensation, the nucleus was fragmented and densely) stained were observed. **b**. BM-1197 induces a dose- and time-dependent apoptosis in OCI-ly8 cellsdetected by Annexin V/PI staining and analyzed using flow cytometry. **c**. Quantification of the apoptotic rate after BM-1197 treatment with increasing dose. **d**. Quantification of the apoptotic rate after BM-1197 treatment with increasing time. **e**. After BM-1197 treatment at indicated time points, the change of Bcl-2 family proteins and apoptotic proteins. **f**. After BM-1197 treatment at indicated dose, the change of Bcl-2 family proteins and apoptotic proteins

To investigate whether BM-1197 can induce cell apoptosis, OCI-ly8 cells were treated with different concentrations of BM-1197 for 24 h and stained with Hoechst 33258. After treatment with different concentrations of BM-1197, apoptotic bodies with intense blue fluorescence were observed (Fig. 2a). In addition, in order to examine whether BM-1197 induced cell apoptosis in a concentration-dependent manner, OCI-ly8 cells were treated with BM-1197 at the indicated dose for 24 h, or with 2 μM BM-1197 for 0, 1, 3, 6, 12 and 24 h, and then double-stained with Annexin V/PI and analyzed by flow cytometry. As seen in Fig. 2b, c, d, the number of early apoptotic cell populations increased with increasing drug concentration and time. Furthermore, OCI-ly8 cells were treated with different concentrations (0, 0.25, 0.5, 1 and 2 μM) of BM-1197 for 24 h or at the same concentration (2 μM) at different time points (1, 3, 6, 12 and 24 h) and then analyzed by western blotting to detect the effect of BM-1197 on changes in the expression of apoptosis pathway-related proteins. After BM-1197 treatment, the expression of cleaved PARP- 1, caspase-3, − 7, and − 9 in OCI-ly8 cells increased in a concentration- and time-dependent manner (Fig. 2e and f). Additionally, BM-1197 can increase the expression of the PUMA protein, which can activate Bax more strongly and change its conformation. We also found that BM-1197 can promote Bax protein conformational changes and the induction of apoptosis in a concentration- and time-dependent manner.

### BM-1197 induces apoptosis in a mitochondrial pathway

**Fig. 3:**
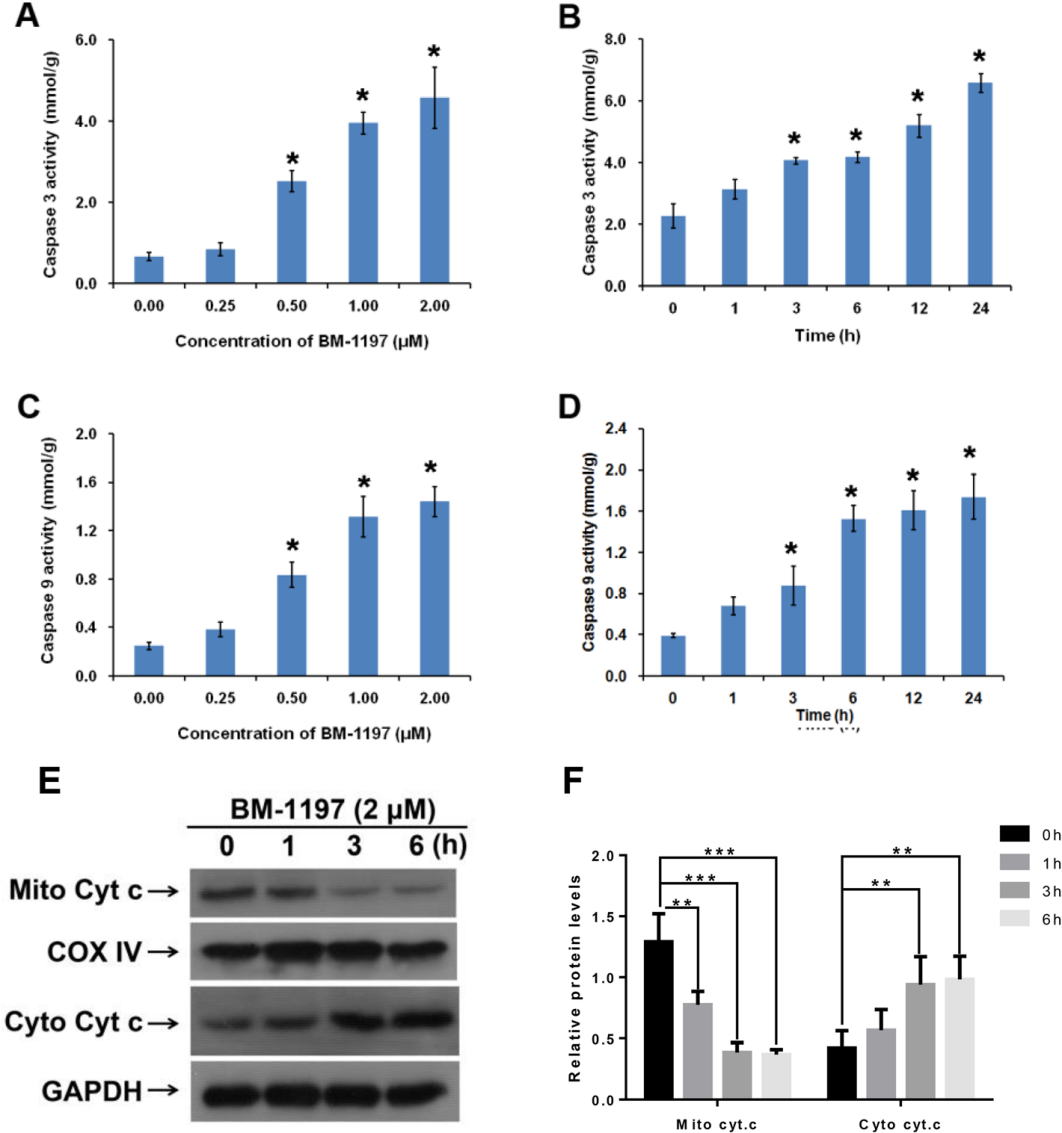
BM-1197 induces apoptosis in a mitochondrial pathway. **a**. Caspase 3 activity in OCI-ly8 cells were measured 24 h after adding BM-1197 and were plotted relative to the DMSO control. **b**. Caspase 3 activity in OCI-ly8 cells were measured in indicated time points after adding BM-1197 and ere plotted relative to the DMSO control. **c**. Caspase 9 activity in OCI-ly8 cells was measured 24 h after treated with BM-1197 and was plotted relative to the DMSO control. **d**. Caspase 9 activity in OCI-ly8 cells was measured in indicated time points after adding BM-1197 and was plotted relative to the DMSO control. **e**. Cytochrome c (Cyt. c) expression in OCI-LY8 mitochondrial and cytosolic fractions were determined by western blot in indicated time points after adding BM-1197. COX IV and GAPDH serve as protein loading controls for the mitochondria and cytosol, respectively. **f**. Quantification of Cytochrome c (Cyt. c) abundance in OCI-LY8 mitochondrial and cytosolic fractions

We next want to clarify whether the anti-tumor effect induced by BM-1197 was associated with the mitochondrial pathway. First, we determined the activities of caspase-3 and caspase-9 in OCI-ly8 cells treated with BM-1197. After treatment, caspase-3 activity (Fig. 3a and b) showed a significant dose- and time-dependent increase. In addition, the activity of caspase-9 also exhibited the same significant changes as caspase-3 (Fig. 3c and d). We then observed rapid release of cytochrome c after treatment with BM-1197 (Fig. 3e and f). In summary, BM-1197 induced NHL cell death via activating the mitochondrial apoptotic pathway.

### Decreasing the expression of mcl-1 enhances the activity of BM-1197

**Fig. 4:**
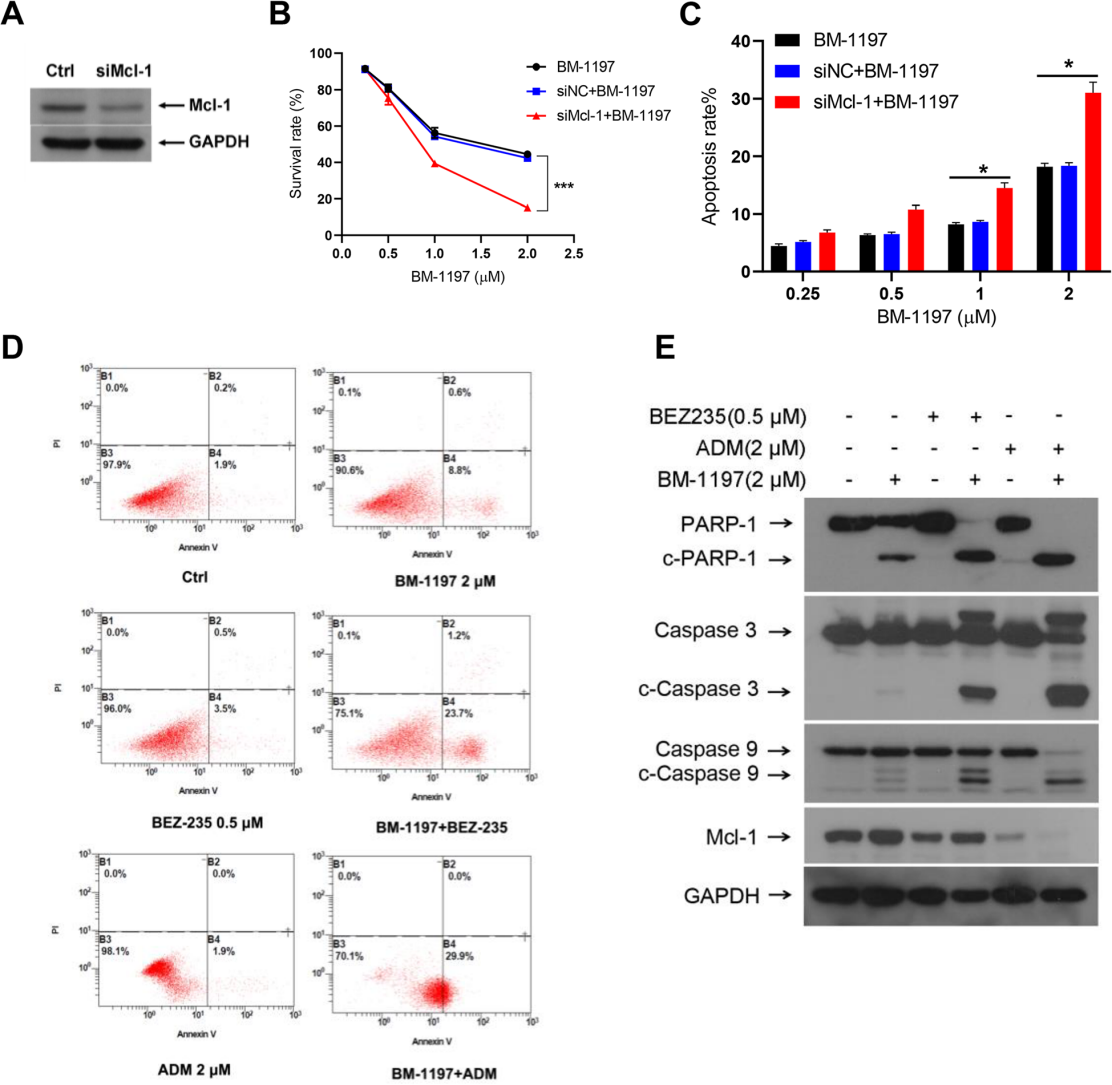
Deregulated the expression of Mcl-1 enhances the activity of BM-1197. **a**. Western blot confirms Mcl-1 knockdown in OCI-ly8 cells co-transfected with targeting or non-targeting (NT) siRNA. **b**. Knockdown of Mcl-1 enhances OCI-ly8 cells to BM-1197 treatment. **c**. BM-1197 induces more apoptosis in Mcl-1 knockdown OCI-ly8 cells. **d**. BM-1197 combined with BEZ235 and ADM can synergistically induce more significant apoptosis detected by flow cytometry. **e**. The expression change of apoptotic proteins and Mcl-1 in OCI-ly8 cells treated with BM-1197, BEZ235, ADM or co-treatment of above drugs

We also want to know whether Mcl-1 was another factor limiting the efficacy of BM-1197 in NHL. We first knocked out the expression of Mcl-1 in OCI-ly8 cells using siRNA (Fig. 4a). As shown in Fig. 4b, the cell viability of normal OCI-ly8 cells or siNC group treated with BM-1197 were similar. However, a significantly decrease in cell viability was observed in the siMcl-1 group after BM-1197 treatment compared to normal control and siNC groups. We further found that with increasing doses of BM-1197, the apoptosis ratio was upregulated in the siMcl-1 group (Fig. 4c). Secondly, we combined BM-1197 with BEZ-235 or ADM, which possess the ability to down-regulate the Mcl-1 expression, to further explore a rational combination regimen. We observed that BM-1197 in combination with BEZ-235 or ADM induced more apoptosis than monotherapy (Fig. 4d). We also discovered that BM-1197 combined with BEZ-235 or ADM upregulated the proteins expression of c-PARP-1, cleaved caspase3 and cleaved caspase9, while significantly down-regulated Mcl-1 expression compared to the single drug or DMSO control group (Fig. 4e).

### BM-1197 exerts anti-tumor function by regulating the interaction between Bcl-2 family proteins

**Fig. 5:**
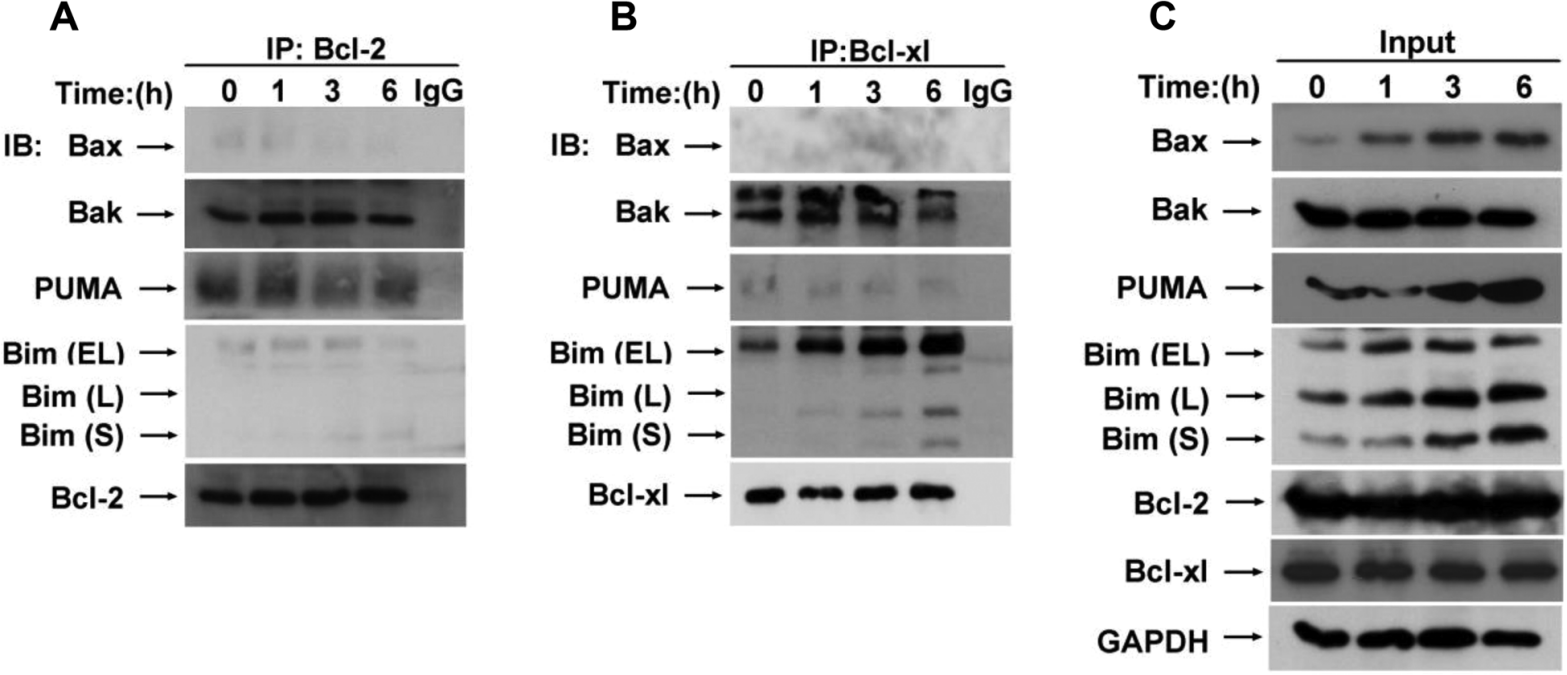
BM-1197 exerts anti-tumor function by regulating the interaction between Bcl-2 family proteins. **a**. Cell lysates were subjected to co-IP using anti-Bcl-2 antibodies, followed by IB to detect the level of Bax, Bak, PUMA, Bim after treated with BM-1197. **b**. Cell lysates were subjected to co-IP using anti-Bcl-xl antibodies, followed by IB to detect the level of Bax, Bak, PUMA, Bim after treated with BM-1197. **c**. The input expression of Bcl-2 family protein after treated with BM-1197 in indicated time

To further investigate the mechanism of action of BM-1197, we used co-immunoprecipitation to detect the interaction of Bcl-2 and Bcl-xl with pro-apoptotic proteins Bax, Bak, Puma and Bim. Our study found that after treatment with BM-1197, the binding of Bax to Bcl-2 was very few and the conjunction of Bax was reduced (Fig. 5a). We also observed that BM-1197 had no effect on the binding of Bcl-2 to Bak. However, after the treatment of BM-1197, the binding of Bak to Bcl-xl was decreased (Fig. 5b). Besides, the binding of Bim to Bcl-2 and Bcl-xl was significantly increased after treatment. The binding of PUMA to Bcl-2 was also reduced, while the binding of PUMA to Bcl-xl was not detected. After treatment of BM-1197, the protein expression of Bax, PUMA and Bim increased rapidly with 1 h (Fig. 5c).

### BM-1197 exerted a strong anti-tumor effect in OCI-Ly8 xenograft models

**Fig. 6:**
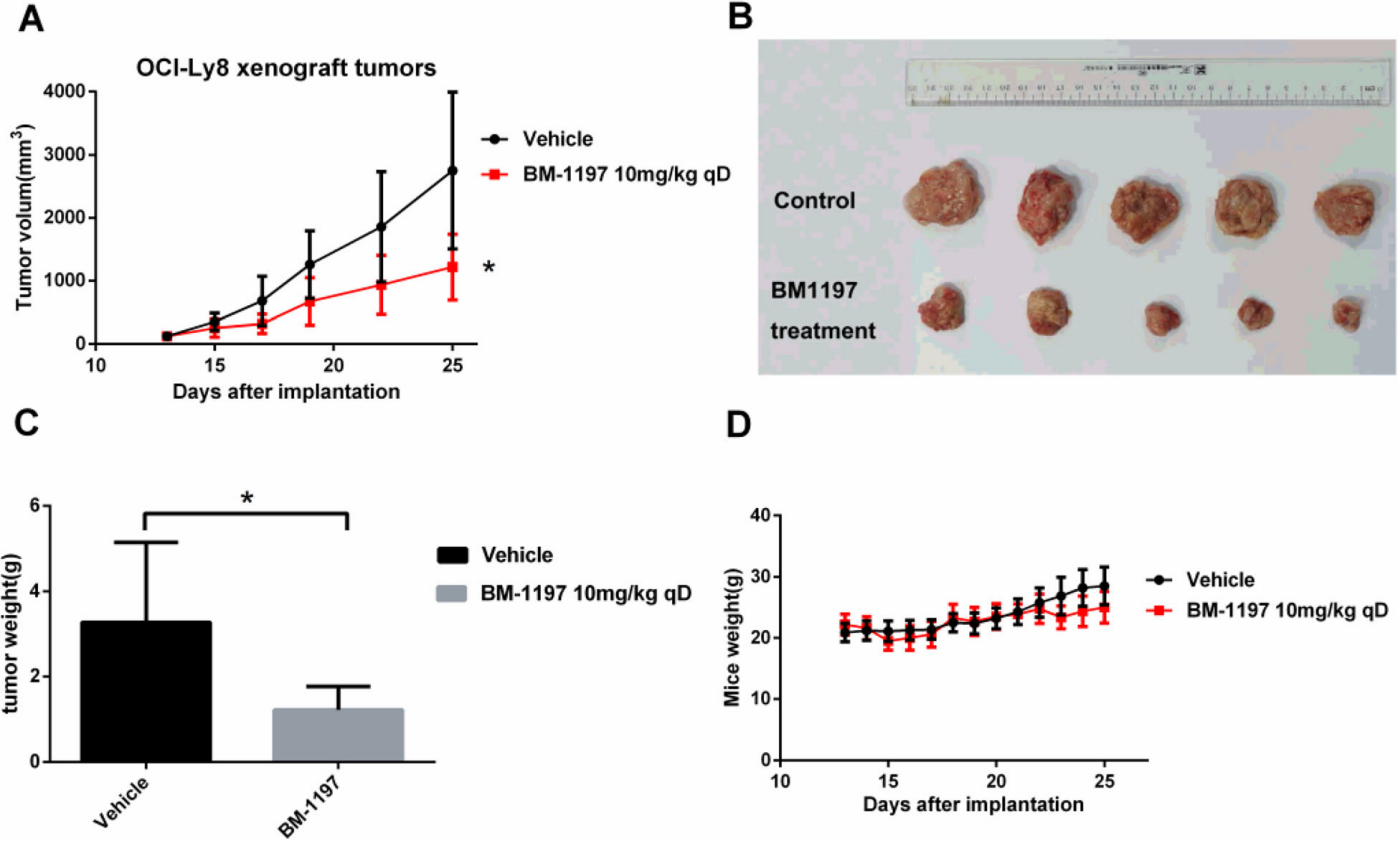
BM-1197 exerted strong anti-tumor effect in OCI-Ly8 xenograft models. **a**. Tumor volumes were measured at indicated days. Compared with control group, BM-1197 could inhibit OCI-Ly8 subcutaneous xenografts tumor growth significantly. Data are shown as mean ± S.E. of five mice in each group. ***: *P* < 0.001. **b**. OCI-Ly8 cells were injected into NOD/SCID mice as indicated. After treated with BM-1197 12 days, mice were sacrificed to harvest tumors. **c**. The tumor weight was supressed by BM-1197 significantly compared with vehicle group. Data are shown as mean ± S.E. of five mice in each group. *: *P* < 0.05. **d**. BM-1197 was well tolerated in those mice since no obvious weight loss upon treatment

The in vivo antitumor effect of BM-1197 was estimated in OCI-Ly8 tumor-bearing mice. Administration of BM-1197 by tail intravenous injection significantly inhibited the tumor growth of OCI-Ly8 subcutaneous xenograft. Compared with an average volume of 2750.7 ± 1245.4 mm^3^ in control vehicle group, the inhibitory effect of 10 mg/kg BM-1197 every day for 12 consecutive days on tumor was 58.36% (Fig. 6a and b). The above results were also consistent with that of tumor weight assessment performed at the end of the experiment (Fig. 6c). BM-1197 was well tolerated in mice because the mice were in good condition, the hair was smooth, and the body weight was not significantly reduced after treatment (Fig. 6d).

## Discussion

In the past few decades, a series of BH3-mimetic small molecules that bind to the BH3 binding sites of anti-apoptotic proteins such as Bcl-2 have been developed. Preclinical studies have shown that the Bcl-2/Bcl-xl dual target inhibitor ABT-737 significantly enhances the anti-tumor effects of chemotherapy and radiotherapy and is effective in a variety of hematological and solid tumors. However, ABT-737 is not able to bind to Mcl-1, which results in tumor drug resistance [13]. Although AT-101 and Obatoclax have entered Phase II clinical trials, they cause severe side effects, and their similarity to BH3 is very poor [14, 15], which promoted the production of the highly selective Bcl-2 inhibitor venetoclax. Venetoclax is approved in the United States for the treatment of patients with chronic lymphocytic leukemia (CLL) with 17p deletion who have received at least one prior therapy [16]. Venetoclax also has shown potent activity against follicular lymphoma (FL), DLBCL, and mantle cell lymphoma (MCL) cell lines [17]. Overall, Bcl-2 may be an effective target to reduce the tumorigenicity of DLBCL.

After ABT-199 was awarded as ‘Breakthrough Therapy Designation’ by the US Food and Drug Administration (FDA) in recognition of its prospects for treatment of patients with chemotherapy- resistant CLL, Bcl-2 inhibitors become a major research area. In this study, we investigated the antitumor effect and mechanism of BM-1197, a potent Bcl-2 and Bcl-xl dual inhibitor on NHL cells both in vitro and in vivo. BM-1197 induces the conformational alteration of Bax protein by blocking the interactions between anti- and pro-apoptotic proteins, induces Bax activation, releases cytochrome c, activates caspase − 9, − 3, − 7, and finally induces NHL cell lines apoptosis.

Overexpression of Bcl-2 leads to abnormal apoptosis and cancer development. A large proportion of follicular lymphomas with Bcl-2 overexpression are transformed into DLBCL. Bcl-xl is also the key regulator of apoptosis in lymphoma cells. Downregulation of Bcl-xl expression induced lymphoma cell apoptosis in follicular lymphoma cells with high expression of Bcl-2 and Bcl-xl [18]. Thus, we first detected the expression of Bcl-2 and Bcl-x in all lymphoma cell lines. Bcl-2 protein is highly expressed in DLBCL cell lines and low in Burkitt lymphoma cell lines and Jurkat cell lines. Bcl-xl protein is highly expressed in all nine cells. In vitro anti-proliferation assay showed that BM-1197 is more sensitive to cells with Bcl-2 overexpression than those with Bcl-2 low expression. Furthermore, the expression of p-Bcl-2 is related to the activity of BM-1197.

Combined use of targeted drugs and chemotherapeutic drugs is an optimal choice for treatment. To this end, we evaluated the effect of the combined use of BM-1197 and three commonly used chemotherapeutic drugs, doxorubicin, vincristine and gemcitabine. Using different proportions of combinations, we calculated the combined index using CompuSyn software developed by Tingchao Chou, which is commonly used in the field [19]. The synergistic effect of BM-1197 and vincristine was the strongest. The synergistic effect was observed with adriamycin in certain proportions. The synergistic effect with gemcitabine was not very obvious. This result provided us a reference for future options using combination therapy.

As a BH3 domain mimetic, BM-1197 mainly affects the activity of the Bcl-2 core family protein of the endogenous apoptotic pathway. Therefore, we have confirmed and verified the BM-1197-induced apoptosis of OCI-ly8 cells in several aspects. First, the appearance of apoptotic sub-G1 population was detected by a simple PI staining method, and Hoechst 33258 staining was used to detect apoptotic bodies from cell morphology. Then Annexin V/PI double staining and flow cytometric analysis was performed to detect apoptotic cells. These three experimental results confirmed that BM-1197 could induce apoptosis in OCI-ly8 cells.

Apoptosis occurs through the exogenous death receptor signaling pathway and the endogenous mitochondrial signaling pathway. Caspase-2, − 8, and − 10 are activated in the death receptor signaling pathway [20]. The endogenous signaling pathway caused the release of cytochrome c from mitochondria that mediated caspase-9 activation. The activation of caspase-9 activated caspase-3 and caspase-7. Activated caspase-3 and caspase-7 degrade large amounts of cellular proteins, leading to apoptosis [21]. It can be seen that the caspase family proteins are important in the apoptotic process. Among them, caspase-3 is the most important terminal cleavage enzyme in the process of cell apoptosis. The main substrate of caspase-3 is poly (ADP-ribose) polymerase (PARP) m which is important for DNA repair and maintenance of genetic integrity. When OCI-ly8 cells were treated with BM-1197 in different concentrations and at different time points, we observed the increased expression of cleaved caspase-9, caspase-7, caspase-3, and PARP-1. This result indicated that BM-1197 induces apoptosis through the endogenous pathway.

In addition to the apoptotic caspase family proteins and their substrates, BM-1197 also promotes apoptosis by upregulating the expression of the PUMA protein. PUMA (p53 upregulated modulator of apoptosis) is a pro-apoptotic gene identified in colon cancer cells in 2001. It was named because it was induced by p53 and has a strong pro-apoptotic function [22, 23]. PUMA is a BH3-only protein, located in the mitochondrial outer membrane. PUMA can directly or indirectly activate Bax/Bak to promote cell apoptosis [24]. BM-1197 upregulated the protein expression of PUMA and then activated Bax/Bak, leading to the occurrence of apoptosis.

The pro-apoptotic proteins Bax and Bak are the promoters of cell apoptosis. Bax and Bak form a polymer in the mitochondrial outer membrane, leading to the release of cytochrome C from the mitochondria into the cytosol. Cytochrome C binds to Apaf-1 and activates caspase-9. Caspase-9 activates caspase-3 and thereby activates the entire caspase cascade to initiate apoptosis. Bcl-2 binds to Bax to form a protein dimer to prevent the activation of Bax, thereby preventing the occurrence of apoptosis. Bcl-2 can also induce the expression of Mcl-1, which forms a dimer with Bak, preventing Bak activation. The BH3-only protein is a pivotal regulator of the activity of the Bcl-2 family and is capable of competing for binding to Bcl-2-like proteins to release Bax/Bak from Bcl-2/Mcl-1 dimers or to directly activate Bax/Bak, promoting apoptosis. BM-1197 does not affect the expression of anti-apoptotic proteins such as Bcl-2, Bcl-xl and Mcl-1. Instead, it upregulates the expression and activity of pro-apoptotic proteins to initiate apoptosis. Therefore, we speculate that BM-1197 can directly bind to Bcl-2/Bcl-xl or upregulate the expression of PUMA protein in OCI-ly8 cells, leading to conformational change of Bax, or promote Bcl-2/Bcl-xl, which leads to the translocation of the Bax protein, and promote the release of cytochrome c, thereby activating caspase-9, followed by activation of caspase-3 and 7. Activation of caspase-3 and -7 leads to cleavage of PARP-1 and cell apoptosis. BM-1197 also exerted a strong tumor-suppression effect in an in vivo study, which may provide another effective way to use this therapeutic drug in the treatment of NHL lymphoma.

Acquired and inherent drug resistance is always a potential concern of target drugs. To develop biomarkers to predict patient responses and stratify patients, or to design combination regimens with increased efficacy and applicability, rational drug development must anticipate potential mechanisms of resistance, both intrinsic and acquired. PI3K could achieve transcriptional, translational, and posttranslational regulation of Bcl-2 family proteins by regulating mTOR, GSK3, FOXO and NF-κB [25, 26]. Meanwhile, Bcl-2 inhibitor resistance may occur via the upregulation of anti-apoptotic proteins such as Bcl-xL and Mcl-1. Our data reveal that downregulation of Mcl-1 or combination with a PI3K inhibitor, BEZ235, could improve the efficacy of BM-1197 in lymphoid malignancies. The synergy of these two drugs may be affected by the PI3K/AKT pathway. Lan V has reported that intrinsic venetoclax-resistant cells possess high AKT activation and are highly sensitive to PI3K/AKT inhibition [27].

## Conclusion

In conclusion, BM-1197 has strong anti-tumor activity against malignant lymphoma cells both in vitro and in vivo. The mechanism of action of BM-1197 is to induce apoptosis through an endogenous apoptotic pathway. In addition, our study supports the rationale for a clinical evaluation of BM-1197 plus PI3K/AKT inhibitor to improve the efficacy of BM-1197. The preclinical activity and mechanism of action of BM-1197 in malignant lymphoma suggests it has the potential to treat malignant lymphoma.

## Data Availability

All data in our study are included in this published article.

## Abbreviations

CCK-8: Cell Counting Kit-8
CI: combined index
CLL: Chronic lymphocytic leukemia
DLBCL: Diffuse large B-cell lymphoma
FL: Follicular lymphoma
MCL: Mantle cell lymphoma
PFS: Progress Free Survival
